# Development of a macromolecular prodrug for the treatment of inflammatory arthritis: mechanisms involved in arthrotropism and sustained therapeutic efficacy

**DOI:** 10.1186/ar3130

**Published:** 2010-09-13

**Authors:** Ling-dong Quan, P Edward Purdue, Xin-ming Liu, Michael D Boska, Subodh M Lele, Geoffrey M Thiele, Ted R Mikuls, Huanyu Dou, Steven R Goldring, Dong Wang

**Affiliations:** 1 Department of Pharmaceutical Sciences, University of Nebraska Medical Center, 986025 Nebraska Medical Center, Omaha, NE 68198-6025, USA; 2 Hospital for Special Surgery, 535 East 70th Street, New York, NY 10021, USA; 3 Department of Radiology, University of Nebraska Medical Center, Omaha, NE 68198-1045, USA; 4 Department of Pathology and Microbiology, University of Nebraska Medical Center, Omaha, NE 68198-3135, USA; 5 Department of Internal Medicine, Section of Rheumatology, University of Nebraska Medical Center, Omaha, NE 68198-3332, USA; 6 Omaha VA Medical Center, 4101 Woolworth Avenue, Omaha, NE 68105, USA; 7 Department of Pharmacology and Experimental Neuroscience, University of Nebraska Medical Center, Omaha, NE 68198-5800, USA; 8 Department of Biomedical Sciences, Texas Tech University Health Sciences Center (TTUHSC), Paul L. Foster School of Medicine, 5001 El Paso Drive, COE for Infectious Diseases, MSB1, Room 4111, El Paso, TX 79905-2827, USA

## Abstract

**Introduction:**

The purpose of the present manuscript is to test the hypothesis that arthrotropic localization and synovial cell internalization account for the unique capacity of *N*-(2-hydroxypropyl)methacrylamide (HPMA) copolymer-dexamethasone conjugate (P-Dex, a macromolecular prodrug of dexamethasone) to induce sustained amelioration of joint inflammation and inhibition of tissue damage in an animal model of inflammatory arthritis.

**Methods:**

Rats with adjuvant-induced arthritis (AA) were treated with P-Dex, free dexamethasone, saline or HPMA homopolymer. To define the biodistribution of P-Dex, conjugates with different imaging labels were given to AA rats and analyzed. Isolated joint tissues were evaluated by fluorescence-activated cell sorting (FACS) and immunohistochemical staining. Cellular uptake of P-Dex and its effects on apoptosis and production of proinflammatory cytokines were examined using human monocyte-macrophages and fibroblasts.

**Results:**

A single systemic administration of P-Dex completely suppressed AA for >20 days. Magnetic resonance imaging demonstrated higher HPMA copolymer influx into the inflamed joints than the normal joints. Immunohistochemistry and FACS analyses of arthritic joints revealed extensive uptake of the polymer conjugate by synovial fibroblasts and myeloid lineage cells. The capacity of P-Dex to suppress inflammation was confirmed in monocyte-macrophage cultures in which P-Dex treatment resulted in suppression of lipopolysaccharide-induced IL-6 and TNFα release. Similarly, TNFα-induced expression of matrix metalloproteinases (MMP1 and MMP3) in synovial fibroblasts from a rheumatoid arthritis patient was suppressed by P-Dex. P-Dex showed no detectable effect on monocyte apoptosis.

**Conclusions:**

P-Dex provides superior and sustained amelioration of AA compared with an equivalent dose of free dexamethasone. The arthrotropism and local retention of P-Dex is attributed to the enhanced vascular permeability in arthritic joints and the internalization of P-Dex by synovial cells. The uptake and processing of P-Dex by macrophages and fibroblasts, and downregulation of proinflammatory mediators, provides an explanation for the sustained anti-inflammatory efficacy of P-Dex in this model of inflammatory arthritis.

## Introduction

Therapeutic strategies targeting cellular components of the inflamed synovial tissue and blockage of specific inflammatory mediators have been shown to be efficacious in ameliorating inflammation and inhibiting joint destruction in patients with rheumatoid arthritis (RA) [1,2]. From a pharmacodynamic perspective, the treatment efficacy mainly depends upon two factors: the specificity of the drug to its molecular target, and the local concentration of the drug where it interacts with its putative target. While most of the efforts to improve RA treatment have been focused on the discovery of agents that target specific molecules or pathways and more potent therapeutic agents, the approach to manipulate the local drug concentration in the synovium and consequently potentiate the efficacy of a particular therapy has been very limited [3-5].

In the past decade, several groups have developed liposomal and protein-based formulations to facilitate the targeting of drugs to arthritic joints [6-9]. Recently, our team has developed the acid-labile arthrotropic macromolecular dexamethasone prodrug based on *N*-(2-hydroxypropyl)methacrylamide (HPMA) copolymer (P-Dex) [10] and has provided preliminary evidence demonstrating its superior anti-inflammatory efficacy compared with an equivalent dose of free dexamethasone (Dex) [11-13]. The present studies were undertaken to delineate the mechanisms involved in the arthrotropism and joint retention of P-Dex, and its capacity to produce sustained amelioration of inflammatory arthritis.

## Materials and methods

### Treatment of adjuvant-induced arthritis rats with P-Dex

Adjuvant-induced arthritis (AA) rats were induced as described previously [11]. On day 14 post induction, the rats were randomized into five groups (eight rats/group): P-Dex half-dose (equivalent Dex dose = 5 mg/kg, single intravenous (i.v.) injection), P-Dex (equivalent Dex dose = 10 mg/kg, single i.v. injection) [13], free Dex (total dose = 10 mg/kg, four intraperitoneal injections, days 14 to 17), saline (single i.v. injection) and HPMA polymer without Dex (PHPMA, single i.v. injection; amount of polymer used is equivalent to P-Dex).

Arthritis flare of the P-Dex group was set as the experimental endpoint, at which time the hind limbs were isolated for bone mineral density (BMD) and histology evaluations. The BMD was measured from the distal tibia to the phalanges of the paw using a pDEXA^® ^Sabre™ X-ray bone densitometer (Norland Medical System, Inc., Fort Atkinson, WI, USA).

All animal experiments were performed according to a protocol approved by the University of Nebraska Medical Center Institutional Animal Care and Use Committee.

### Clinical measurements

The articular index score was measured during the treatment as described previously [13]. The scores were applied to each hind limb by the same observer (LDQ) from day 8 to day 34 post-arthritis induction, and the sum of the two hind limb scores for each animal were recorded. The ankle diameter (medial to lateral) was measured using a digital caliper (World Prescision Instruments, Inc., Saraspta, FL, USA).

### Histological analysis

The isolated hind limbs were fixed with buffered formalin and were decalcified. Thin sections (5 μm) were cut approximately 200 μm apart and were H & E stained. The joints were histologically graded by a pathologist (SML), who was blind to the treatment groups, using a scoring system adapted from previous work [13]. Each histopathologic feature was graded as follows: synovial cell lining hyperplasia (0 to 2); pannus formation (0 to 3); mononuclear cell infiltration (0 to 3); polymorphonuclear leukocytes infiltration in periarticular soft tissue (0 to 3); cellular infiltration and bone erosion at distal tibia (0 to 3); and cellular infiltration of cartilage (0 to 2). The score for every histopathologic feature was summed for each animal.

### Quantitative analysis of joint vascular leakage using magnetic resonance imaging

High-resolution T_1_-weighted magnetic resonance imaging (MRI) scans and T_1 _maps were acquired before and after (every 10 minutes for 4 hours) the injection of DOTA-Gd^3+^-labeled HPMA copolymer (P-DOTA-Gd^3+^) [11]. All MRI scans were performed on a Bruker Avance MRI and spectroscopy system (7T/21 cm; Bruker, Karlsruhe, Germany). T_1 _maps were acquired using a Look-Locker technique [14]. Patlak plots were used to estimate the tissue transfer constant of P-DOTA-Gd^3+ ^(*K_i_*). T_1 _and *K_i _*maps were reconstructed using the neuropipes software suite (a software suite written in 'C' for the apodization, reconstruction, and curve-fitting of MRI images. Developed by Dr. James Ewing at Henry Ford Hospital, Detroit, MI, USA). Both unidirectional and bidirectional transfer models were used in the analyses. F-tests were performed between the two models, and each model was tested against no leakage on a pixel-by-pixel basis [15].

### Biodistribution of ^125^I -labeled P-Dex

Tyrosine amide-containing P-Dex (P-Dex-Tyr-NH_2_) was iodinated using a standard chloramine T method [16]. For the biodistribution study, ^125^I-labeled P-Dex-Tyr-NH_2 _(mixed with P-Dex-Tyr-NH_2 _without ^125^I, Dex equivalent dose = 5 mg/kg) was administered to AA rats and healthy rats (six rats/group) via tail vein injection. The animals were euthanized 24 hours post administration. Major organs and tissues were collected and evaluated with a γ-counter (Minaxi Auto-gamma 5000 series; Packard Instrument Company, Meriden, CT, USA).

### Immunohistochemical analysis of arthritic joints

In the MRI studies, HPMA copolymer labeled with Alexa Fluor^® ^488 (P-Alexa) was given to AA rats simultaneously with P-DOTA-Gd^3+^. At 24 hours post injection, hind limbs were isolated and fixed with 0.5% paraformaldehyde in PBS and were decalcified with 10% ethylenediamine tetraacetic acid. Tissues were paraffin embedded and sections corresponding to the MRI hot-spots were selected for immunohistochemical analysis. After deparaffinization, the sections were incubated for 30 minutes with citrate buffer (10 mM, pH = 6.0) at 95°C, followed by incubation with 10% goat serum/PBS for 20 minutes at room temperature. After addition of the primary antibodies (CD68 (10 μg/ml) or prolyl 4-hydroxylase (10 μg/ml), diluted in 10% goat serum/PBS), the sections were incubated overnight at 4°C in a humidified chamber. After washing with PBS (three times), diluted phycoerythrin-labeled rabbit anti-mouse IgG secondary antibody (5 μg/ml) was added and incubated for 30 minutes in the dark at room temperature. In control experiments, primary antibodies were replaced by PBS and the samples were processed as described above. The processed tissue sections were then evaluated with confocal microscopy.

### Fluorescence-activated cell sorting analysis of cells isolated from synovial tissue

At 14 days post induction, P-Alexa was given to AA rats by tail vein injection. At 24 hours post injection, ankle joint-associated soft tissues were surgically removed with a scalpel and minced aseptically. The tissues were further digested with collagenase type I (1 mg/ml; Sigma-Aldrich, St Louis, MO, USA) at 37°C for 30 minutes. After passing through a 70 μm cell strainer, a single-cell suspension (2 × 10^6^/ml) was obtained. ACK Lysing Buffer (Quality Biological, Gaithersburg, MD, USA) was then used to remove blood cells.

For FACS evaluation, the cells were first incubated respectively with three primary antibodies - CD68 (1:100 dilution; AbD Serotec, Raleigh, NC, USA), CD11c (1:10 dilution; abcam Inc., Cambridge, MA, USA) and prolyl-4-hydroxylase (1:50 dilution; Acris Antibodies, Herford, Germany) - each for 30 minutes on ice. The cells were then incubated with phycoerythrin-labeled rabbit anti-mouse IgG secondary antibody (1:100 dilution; BD Biosciences, San Jose, CA, USA) for another 30 minutes on ice. Isotype-matched mouse IgG_1 _and mouse IgG_2a _(BD Biosciences) were used as negative controls. Following the final wash, the cells were analyzed with flow cytometry.

### Macrophage and fibroblast cultures

CD14-positive monocytes were prepared from peripheral blood mononuclear cells derived from de-identified normal human donors as described previously [17]. Cells were cultured for 24 hours at a cell density of 10^6^/ml in α-MEM medium (Invitrogen, Carlsbad, CA, USA) supplemented with 10% FBS (VWR, West Chester, PA, USA) and 1% antibiotic/antimycotic (Invitrogen) in the presence of 10 ng/ml human macrophage colony-stimulating factor (Peprotech, Rocky Hill, NJ, USA) in 24-well tissue culture plates (1 ml/well). Cells were then pulsed for 4 hours with P-Dex (5 μM) or Dex (0.6 μM), washed and replenished with fresh medium. The two treatments had equivalent doses of Dex.

After 24 hours, cells were either analyzed by confocal microscopy and flow cytometry or challenged with inflammatory mediators. For confocal microscopy, the lysosomal and nuclear compartments were stained with LysoTracker Red DND-99 and Hoechst 33342 (Invitrogen), respectively. For flow cytometric analysis of apoptosis, cells were analyzed using the Vybrant Apoptosis Assay Kit #3 (Invitrogen) in accordance with the manufacturer's recommendations. For inflammatory challenge, cells were treated with 40 pg/ml lipopolysaccharide (LPS) for 6 hours, after which conditioned media were analyzed for inflammatory cytokine production (that is, TNFα and IL-6) by ELISA (BD Bioscience, San Diego, CA, USA).

Fibroblasts were isolated from periprosthetic tissues retrieved from patients undergoing total hip replacement revision surgery and from synovial membranes of RA patients. After digestion with collagenase (5 mg/ml), fibroblasts were obtained by passing through a 70 μm cell strainer. The cells were subsequently cultured at a density of 5 × 10^4^/ml in α-MEM medium supplemented with 10% FBS and 1% antibiotic/antimycotic in 24-well tissue culture plates (1 ml/well).

Treatment with Dex or P-Dex and confocal microscopy were performed as described above for monocytes. For inflammatory challenge, fibroblasts were incubated for 24 hours with 100 ng/ml human TNFα (Peprotech), following which conditioned media were analyzed for prostaglandin E_2 _by ELISA (BD Bioscience) and total cellular RNA was prepared (RNeasy Mini Kit, QIAGEN, Inc., Valencia, CA, USA) and analyzed by real-time RT-PCR for matrix metalloproteinases (MMP1 and MMP3) expression.

All human cells experiments were approved by the Institutional Review Board of Hospital for Special Surgery.

### Statistical methods

One-way analysis of variance was used in the data analysis, followed by a *post hoc *test (Student-Newman-Keuls) for multiple comparisons using Instant Biostatistics (GraphPad Software, La Jolla, CA, USA). *P *< 0.05 was considered statistically significant.

## Results

### Sustained amelioration of joint inflammation by P-Dex

**Figure 1:**
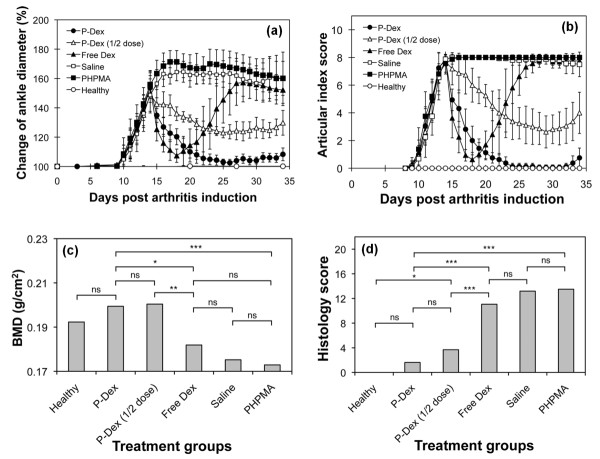
**Treatment of adjuvant-induced arthritis rats with a macromolecular prodrug of dexamethasone**. The treatment of adjuvant-induced arthritis (AA) rats (eight rats/group) with acid-labile *N*-(2-hydroxypropyl)methacrylamide (HPMA) copolymer-dexamethasone conjugate (P-Dex), half-dose P-Dex, free dexamethasone (Dex), HPMA polymer without Dex (PHPMA) and saline were summarized in this figure. An untreated healthy control group was also included. **(a) **Changes in AA rat ankle diameters during the treatment. **(b) **Changes in articular index score of the animals during the treatment. **(c) **Endpoint bone mineral density (BMD) of the ankle joints for all the animal groups. **(d) **Average histology scores of the ankle joints from the six animal groups. ***P < 0.001, **P < 0.01, *P < 0.05; ns, not significant.

As shown in Figure 1a, b, the AA rats respond to free Dex and P-Dex (10 mg/kg) treatment immediately with a dramatic reduction in joint inflammation. For free Dex, a flare in joint inflammation was observed immediately upon cessation of the treatment; and by the end of the study, free Dex, PHPMA and the saline groups all had similar ankle diameter and articular index scores. In contrast, administration of single-dose P-Dex provided a sustained and dose-dependent amelioration of joint inflammation that lasted for 20 days, when minor signs of joint swelling was found in two rats of the P-Dex group.

### Bone mineral density assessment

The saline, PHPMA and free Dex groups had lower mean ankle joint BMD values of 0.17 to 0.18 g/cm^2^, while the P-Dex-treated (10 mg/kg and 5 mg/kg) groups and healthy groups maintain higher mean BMD values of 0.19 to 0.2 g/cm^2^. The statistical analyses indicated that the average BMD values of P-Dex groups (10 mg/kg and 5 mg/kg) were significantly higher (*P *< 0.05) than those of the saline, PHPMA or free Dex-treated groups (Figure 1c).

### Histological evaluation of ankle joints

**Figure 2:**
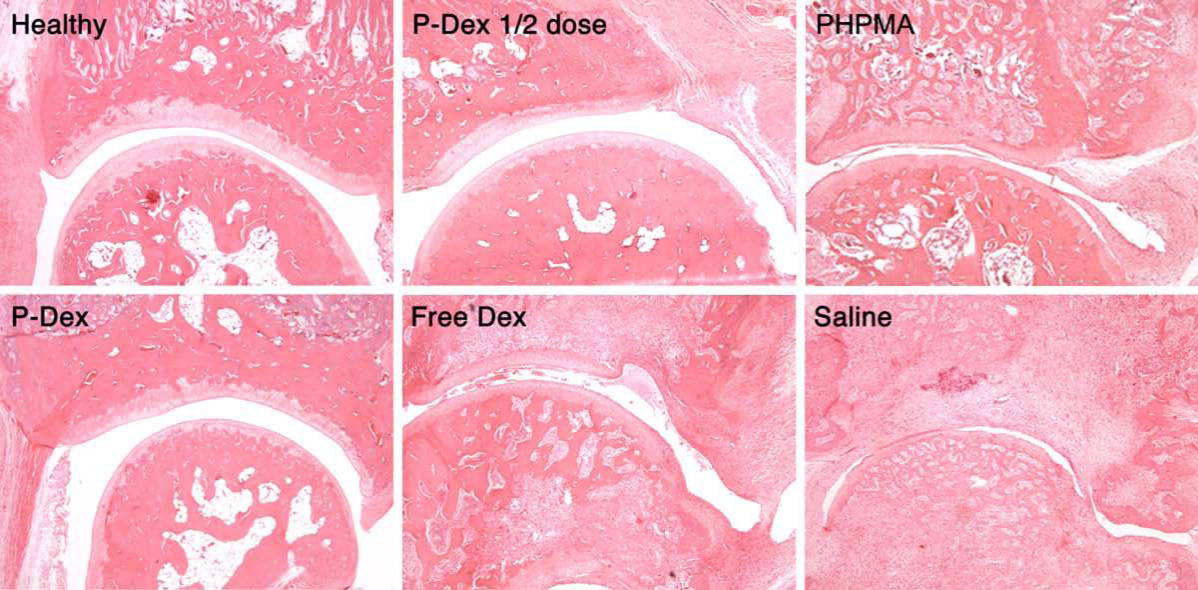
**Light micrographs (magnification, 100x) of ankle joint histology for the six animal groups**. Synovial lining and villous hyperplasia, cellular infiltration in periarticular soft tissue, bone and cartilage destruction are clearly evident in the free dexamethasone (Dex), the *N*-(2-hydroxypropyl)methacrylamide polymer without Dex (PHPMA) and the saline groups. P-Dex, acid-labile *N*-(2-hydroxypropyl)methacrylamide copolymer-dexamethasone conjugate.

As shown in Figure 1d, both P-Dex groups had low average histology scores. No significant difference was found between high-dose P-Dex and the healthy control. The histology scores for the free Dex, saline and PHPMA groups were much worse (> 11). Figure 2 shows that both P-Dex treatments preserve bone and cartilage, with findings similar to the healthy control. Saline and PHPMA groups, however, exhibit the highest scores in each category of the histological grading. Synovial cell lining hyperplasia, pannus formation, polymorphonuclear leukocytes infiltration in periarticular soft tissue, cellular infiltration of cartilage, cellular infiltration and bone destruction at the distal tibia were present in all cases. For the free Dex group, only one out of eight rats was found with a relatively low histology score.

### Quantitative analysis of joint vascular leakage using magnetic resonance imaging

**Figure 3:**
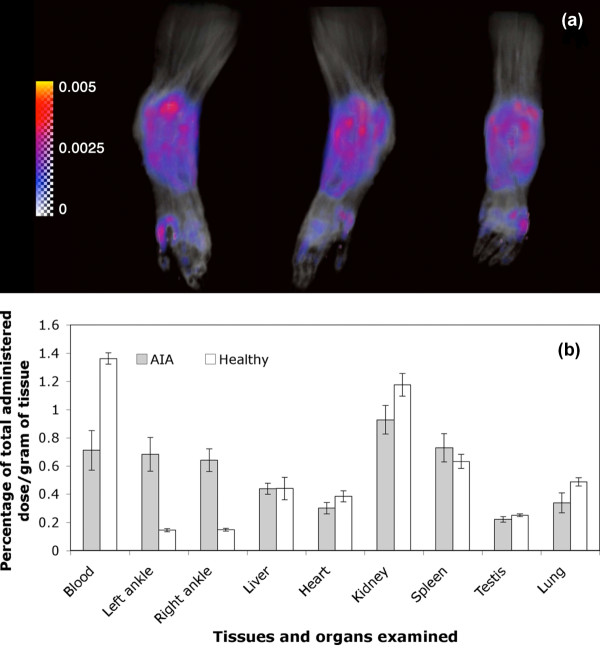
**Arthrotropism of *N*-(2-hydroxypropyl)methacrylamide copolymer conjugates**. **(a) **Maximum intensity projection images (black and white) of a representative adjuvant-induced arthritis (AA) rat right-hind limb from different angles (anterior, lateral and posterior), superimposed with a tissue transfer constant of P-DOTA-Gd^3+ ^(*K_i_*) map (color). Inserted color bar represents the color code for *K_i _*values in the map from 0 to 0.005/min. **(b) **Biodistribution of ^125^I-labeled acid-labile *N*-(2-hydroxypropyl)methacrylamide copolymer-dexamethasone conjugate (P-Dex) in healthy rats (open bars) and in AIA rats (solid bars) at 24 hours post administration.

Using a Patlak plot, a *K_i _*map was constructed and superimposed with the maximum intensity projection image at different angles (Figure 3a; see also Additional file 1 for movie). The region with the highest *K_i _*was associated with the inflamed synovium region close to the distal tibia where there was evidence of severe bone and cartilage erosion. An average *k_i _*value of 0.0032/min was obtained based on evaluation of three AA rats. The *K_i _*for healthy rats was close to zero.

### Biodistribution of P-Dex

Quantitative biodistribution data for ^125^I-labeled P-Dex-Tyr-NH_2 _in both AA and healthy rats were acquired at 24 hours post treatment (Figure 3b). Similar percentages of the injected dose per gram of tissue were found in most tissues isolated from healthy and AA rats except the ankle joints and blood. Arthritic joints showed a significantly higher P-Dex distribution compared with the healthy joints (*P *< 0.0001).

### Immunohistochemical analysis

**Figure 4:**
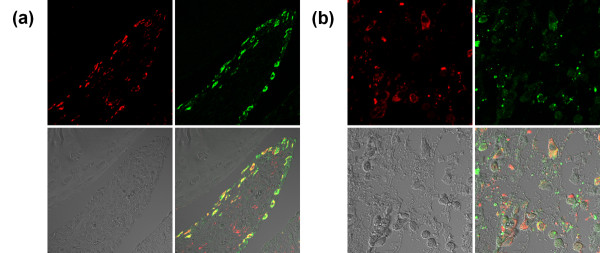
**Immunohistochemical staining of sections from inflamed joints with anti-CD68 or anti-prolyl-4-hydroxylase antibodies**. Representative confocal microscopic images of **(a) **anti-CD68 and **(b) **anti-prolyl-4-hydroxylase immunohistochemical staining of decalcified, Alexa Fluor^® ^488-labeled *N*-(2-hydroxypropyl)methacrylamide copolymer (P-Alexa)-treated adjuvant-induced arthritis (AA) rat ankle joint sections are summarized in this figure. Each panel is composed of four sub-images: antibody red staining, P-Alexa green signal, differential interference contrast (DIC) image and the co-localization of the three. The co-localization of red and green color in both panels yields a yellow color, which confirms the uptake of the HPMA copolymer conjugate by CD68-positive (macrophage-like) or prolyl-4-hydroxylase-positive (fibroblast-like) synoviocytes in AA rat ankle joints.

As shown in Figure 4, there was extensive uptake of P-Alexa by synovial cells with primary localization to intracellular vesicles. Immunohistochemical staining of the sections prepared from inflamed joints with anti-CD68 or anti-prolyl-4-hydroxylase antibodies revealed co-localization of P-Alexa-positive intracellular vesicles with both macrophage-like (CD-68-positive) and fibroblast-like (prolyl-4-hydroxylase-positive) synoviocytes. The double-stained cells could be found throughout the synovium but were particularly abundant within the synovial lining.

### FACS analysis of HPMA copolymer internalization by AA rat synoviocytes

**Figure 5:**
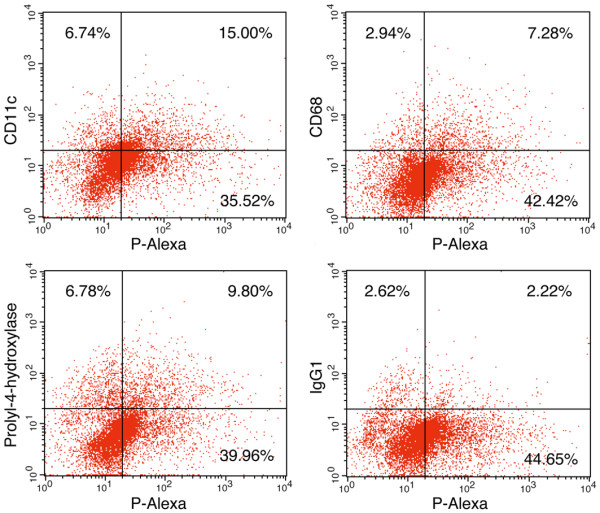
**Fluorescence-activated cell sorting analysis of cells isolated from adjuvant-induced arthritis rat synovial tissue**. Twenty-four hours after systemic administration of *N*-(2-hydroxypropyl)methacrylamide copolymer labeled with Alexa Fluor^® ^488 (P-Alexa), cells were isolated from adjuvant-induced arthritis rat synovial tissue for fluorescence-activated cell sorting (FACS) analysis. IgG_1 _was used as control for CD68 and prolyl-4-hydroxylase. IgG_2a _was used as control for CD11c. The result for IgG_2a _is similar to that for IgG_1 _with the upper right quadrant detected at 2.45% (data not shown).

As shown in Figure 5, FACS analysis revealed that >30% of the cells disaggregated from the AA rat synovial tissue were P-Alexa-positive. The P-Alexa-positive cells included subpopulations of cells that were CD68-positive (5.1%, isotype control corrected), CD11c-positive (12.6%, isotype control corrected) or prolyl-4-hydroxylase-positive (7.6%, isotype control corrected).

### Confocal microscopic analysis of P-Dex internalization and subcellular partitioning by inflammatory cells

**Figure 6:**
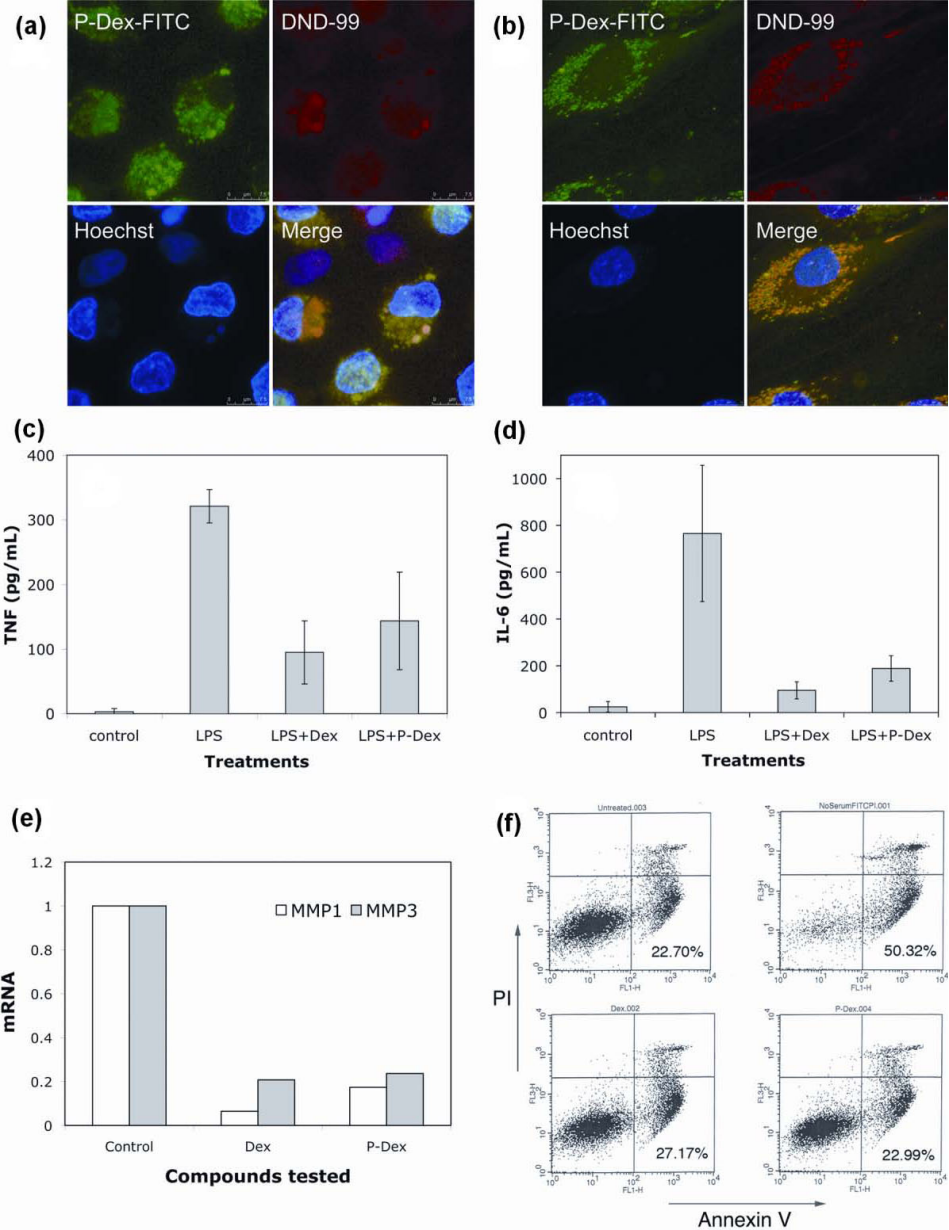
**Internalization and biological effects of acid-labile *N*-(2-hydroxypropyl)methacrylamide copolymer-dexamethasone conjugate in cultured human cells**. Confocal images show the internalization and lysosomal localization of FITC-labeled acid-labile *N*-(2-hydroxypropyl)methacrylamide copolymer-dexamethasone conjugate (P-Dex) by **(a) **cultured human monocytes and **(b) **fibroblasts. In the human monocyte cultures, the internalized P-Dex represses lipopolysaccharide (LPS)-mediated production of **(c) **TNFα and **(d) **IL-6 to levels comparable with those found with free dexamethasone (Dex) (*n *= 3, *P *< 0.0002). **(e) **In synovial fibroblasts from a rheumatoid arthritis patient, P-Dex suppresses TNFα-induced expression of matrix metalloproteinases (MMP1 & MMP3) mRNA. **(f) **Human monocyte apoptosis was detected in untreated monocytes (upper left panel), and was greatly increased after serum withdrawal for 24 hours (upper right panel). Neither Dex (lower left) nor P-Dex (lower right) increased apoptosis above basal levels.

To define the intracellular localization following uptake of P-Dex in macrophage lineage and fibroblastic cells, human monocytes were prepared from peripheral blood and fibroblasts were prepared from the synovial-like tissues of patients undergoing revision surgery for prosthetic loosening after joint replacement. Confocal fluorescence microscopic images (Figure 6a, b), demonstrate co-localization of the FITC-labeled prodrug with the lysosomal marker DND-99 in both cell types. This localization was also maintained during subsequent culturing (not shown).

### P-Dex inhibits LPS or TNFα induced release of proinflammatory products in human monocytes and fibroblasts

To demonstrate the effect of P-Dex on production of proinflammatory mediators, monocytes were preincubated with P-Dex or Dex for 4 hours and then challenged with LPS. As shown in Figure 6c, d, pretreatment with P-Dex or Dex resulted in marked inhibition of LPS-induced TNFα and IL-6 production. Comparable inhibition of TNF-induced expression of MMP1 and MMP3 (Figure 6e) and prostaglandin E_2 _production (data not shown) was also observed in RA synovial fibroblasts pretreated with P-Dex or Dex. Finally, monocyte apoptosis was unchanged from basal levels following uptake of both P-Dex and Dex (Figure 6f).

## Discussion

In preliminary studies, we reported that a single dose of P-Dex (synthesized by either polymer analogues reaction or Dex-containing monomer copolymerization) could result in more potent resolution of joint inflammation compared with free Dex [12,13]. In the current investigation we have extended these observations and established the full time course of sustained anti-inflammatory efficacy for P-Dex (> 20 days), which is accompanied by protection from both bone and cartilage destruction. In addition, we demonstrated the intracellular localization of P-Dex in synovial cells after systemic delivery and established its efficacy in inhibiting proinflammatory cytokine release.

The rationale for the use of the HPMA copolymer as a drug-targeting vehicle is based on observations indicating that synovial inflammation is associated with enhanced vascular permeability to macromolecules [18]. Our MRI data using P-DOTA-Gd^3+ ^quantitatively confirmed the enhanced vascular permeability of the synovial tissues to the HPMA copolymers and provides an explanation for its unique arthrotropism. Of interest, the tissues with the highest *K_i _*values (hot-spots) were in proximity to regions that histologically were associated with the most severe bone and cartilage damage. This suggests that there is a relationship between the quantitative delivery of the polymer-based prodrug and the severity of synovitis, with preferential localization of the prodrug at sites of maximal inflammation. The MRI results were confirmed by biodistribution studies using ^125^I-labeled HPMA copolymer-dexamethasone conjugate (P-Dex-Tye-NH_2_-^125^I). These results provided quantitative affirmation that the distribution of P-Dex at 24 hours post administration was four or five times higher in the AA rat ankle joints compared with healthy joints.

Previous studies have not provided an explanation for the retention of macromolecules at sites of inflammation. To address this issue, we studied the cellular localization of the prodrug within inflamed joints after systemic administration of Alexa Fluor^® ^488-labeled HPMA copolymer (P-Alexa). Histologic analysis of inflamed joints revealed that P-Alexa was internalized by synoviocytes. Immunohistochemical and FACS analyses identified these cells as type A synoviocytes (macrophage-like), type B synoviocytes (fibroblast-like) and dendritic cells. The internalization of different HPMA copolymers by murine macrophages and fibroblasts has been reported previously [19,20]. The internalization and subcellular partitioning of P-Dex, however, have not been evaluated. Our studies utilizing FITC-labeled P-Dex and immunohistochemical staining with the lysozomal marker DND-99 confirmed the co-localization of the macromolecular prodrug in a lysosomal compartment consistent with internalization via an endocytic pathway, and flow cytometric analysis showed that P-Dex uptake did not induce apoptosis. HPMA copolymers such as P-Dex are water-soluble polymers, and thus are restricted by the lysosomal membrane from escaping the endosome/lysosome compartments once internalized by the cells. Within these subcellular vesicles, P-Dex is exposed to an acidic environment (pH ~5.5); and since the Dex is conjugated to the HPMA copolymer via an acid-labile hydrazone bond, the prodrug is subject to gradual hydrolysis and subsequent release of the active drug [12]. The presence of intracellular prodrug activation was confirmed by the capacity of the internalized P-Dex to inhibit TNFα and IL-6 release from LPS-treated macrophages and to reduce expression of MMP1 and MMP3 in RA synovial fibroblasts. Protective effects of P-Dex treatment were maintained for at least 7 days after exposure to cells *in vitro*. We speculate that the sustained therapeutic effect observed in the P-Dex treatment is due to the prolonged residence of P-Dex within the synoviocytes and their gradual low pH-triggered activation within lysosomes, followed by the release of free Dex into the cytosol.

## Conclusions

The data presented in this work support the conclusion that the superior and long-lasting (> 20 days) therapeutic effects of single-dose P-Dex may be attributed to two key factors. First, the enhanced vascular permeability in the inflamed joints facilitates the arthrotropism of the macromolecular prodrug. Second, the dynamic uptake of the prodrug by activated synoviocytes and subsequent trafficking to an acidic lysosomal compartment provides an environment in which there is gradual local prodrug activation and release of active drug resulting in sustained anti-inflammatory signaling through blockade of proinflammatory cytokine production. This is the first report of an enhanced permeability and retention effect of a macromolecular prodrug in an inflammatory disease. Of interest, this retention mechanism is fundamentally different from the enhanced permeability and retention effect associated with solid tumors [21]. The combined capability of time-dependent drug activation/release at sites of disease pathology and selective delivery of therapeutic agents to sites of joint inflammation establishes a novel pathway for the future design and development of therapies for patients with RA and related forms of inflammatory diseases.

## Abbreviations

AA: adjuvant-induced arthritis; BMD: bone mineral density; Dex: dexamethasone; ELISA: enzyme-linked immunosorbent assay; FACS: fluorescence-activated cell sorting; FBS: fetal bovine serum; FITC: fluorescein isothiocyanate; H & E: hematoxylin and eosin; HPMA: *N*-(2-hydroxypropyl)methacrylamide; IL: interleukin; i.v.: intravenous; *K_i_*: tissue transfer constant of P-DOTA-Gd^3+^; LPS: lipopolysaccharide; MEM: modified Eagle's medium; MMP: matrix metalloproteinase; P-Alexa: HPMA copolymer labeled with Alexa Fluor^® ^488; PBS: phosphate-buffered saline; P-Dex: acid-labile HPMA copolymer-dexamethasone conjugate; P-Dex-Tyr-NH_2_: tyrosine amide-containing P-Dex; P-DOTA-Gd^3+^: DOTA-Gd^3+^-labeled HPMA copolymer; PHPMA: HPMA polymer without Dex; RA: rheumatoid arthritis; TNF: tumor necrosis factor.

## Competing interests

As a co-inventor, DW has filed a patent application related to the content of this manuscript. All remaining authors declare that they have no competing interests.

## Authors' contributions

LDQ synthesized the polymer conjugates used in the study, and performed the animal treatment, immunohistochemistry and FACS experiments. He also prepared the first draft of the manuscript. PEP carried out all cell culture studies, and participated in the data interpretation and manuscript preparation. XML synthesized all the monomers and supported LDQ in the conjugate synthesis. MDB performed the MRI analysis and data interpretation. SML performed the histology evaluation. GMT designed the FACS experiments and supported LDQ in FACS data analysis. TRM participated in the data interpretation and manuscript preparation. HD designed the immunohistochemistry experiments and supported LDQ in the data analysis. SRG participated in the general design of the experiments, data interpretation and manuscript preparation. DW conceived the study, and led its design, coordination, data interpretation and manuscript preparation. All authors read and approved the final manuscript.

## Supplementary Material

Additional file 1 **Severe vasculature leakage associated with adjuvant-induced arthritis (AA) rat joint**. A movie demonstrating the tissue transfer constant (*K_i_*) (color) of the HPMA copolymer conjugate labeled with DOTA-Gd^3+ ^(P-DOTA-Gd^3+^) in a representative AA rat right-hind limb, superimposed with a maximum intensity projection (MIP) image (black and white) of the same limb. The inserted color bar represents the color code for *K_i _*values in the map from 0 to 0.005/min. Higher vascular leakage was found to be associated with the soft tissue around the distal tibia. Click here for file
